# Supplementary material for: Defining bottlenecks and opportunities for Lassa virus neutralization by structural profiling of vaccine-induced polyclonal antibody responses
Source: bioRxiv. 2023 Dec 23:2023.12.21.572918. Preprint. [Version 1] doi: 10.1101/2023.12.21.572918 (PMC10769344; doi:10.1101/2023.12.21.572918)
Supplement: Supplement 1 [file media-1.pdf]

Supplemental information

Defining bottlenecks and opportunities for Lassa virus neutralization by structural profiling of vaccine-induced polyclonal antibody responses

Philip J.M. Brouwer, Hailee R. Perrett, Tim Beaumont, Haye Nijhuis, Sabine Kruijer, Judith A. Burger, Wen-Hsin Lee, Helena Müller-Kraüter, Rogier W. Sanders, Thomas Strecker, Marit J. van Gils, Andrew B. Ward

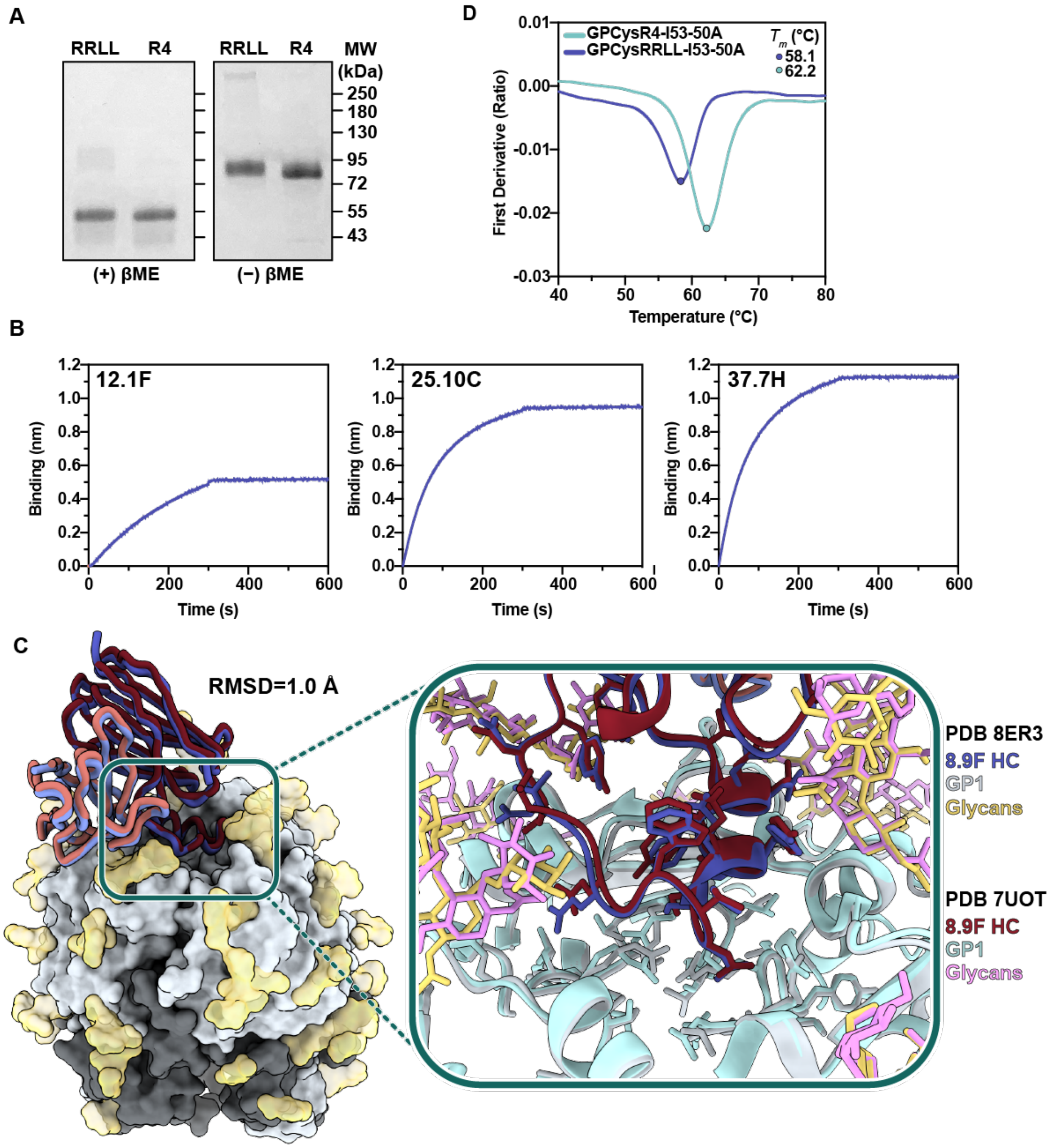

Fig. S1. Characterization and comparison of GPCysRRLL-I53-50A, related to figure 1. (A) SDS-PAGE comparison of cleavage between GPCysRRLL-I53-50A co-transfected with S1P and GPCysR4-I53-50A co-transfected with furin in reducing (left) and non-reducing (right) conditions. **(B)** BLI sensorgrams indicating immobilized 12.1F, 25.10C, and 37.7H IgGs binding to GPCysRRLL-I53-50A at concentration of 120 nM. **(C)** Comparison of GPCysRRLL-I53-50A bound to 8.9F with fully native GPC bound to 8.9F and 37.2D.[1] **(D)** Thermostability of GPCysRRLL-I53-50A and GPCysR4-I53-50A assessed by nanoDSF. Melting temperatures (T_m) are calculated as the inflection point (circles) of the ratio of signal at 350 and 330 nM. Each melting curve is a representative of triplicate curves with T_m within ±0.1°C.

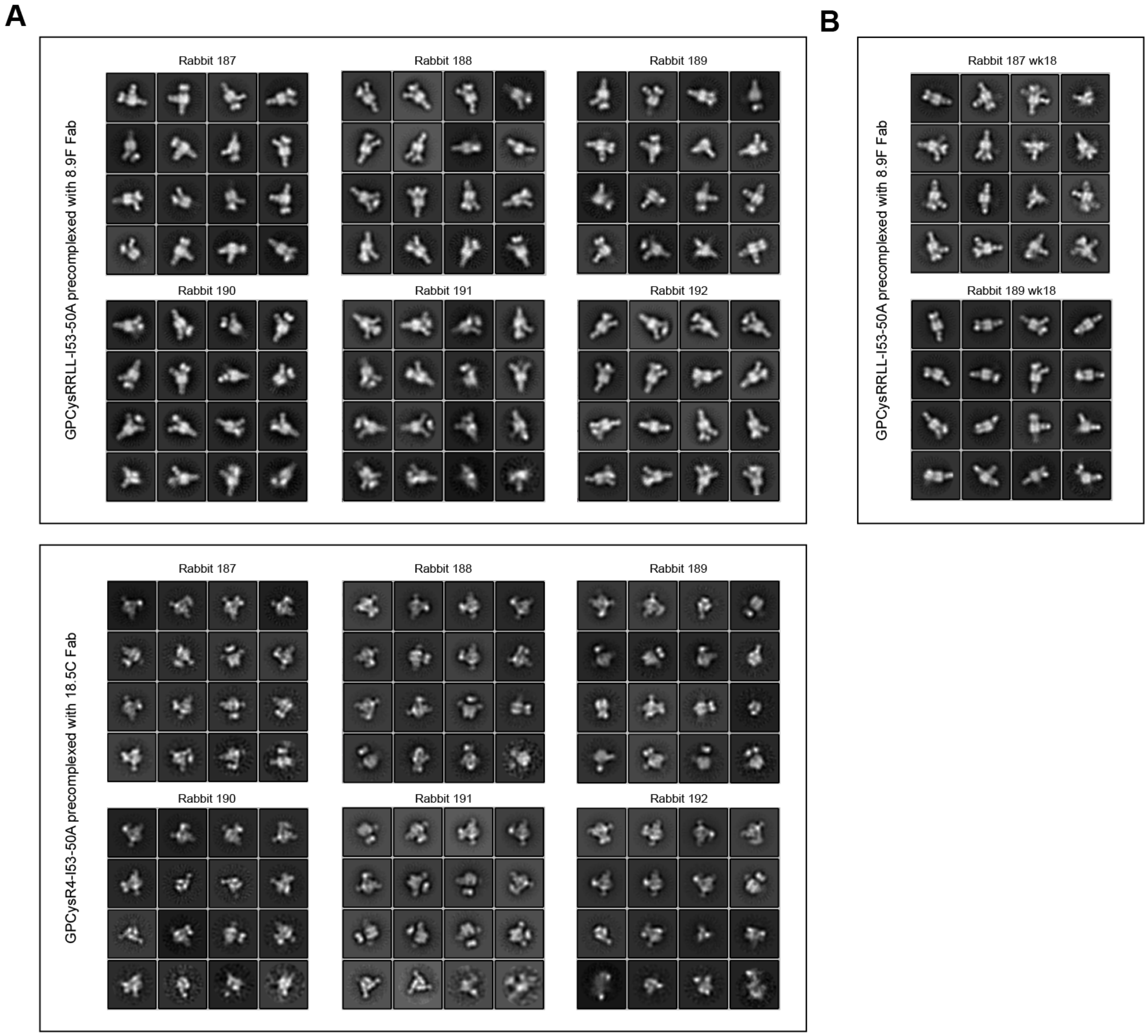

Figure S2. 2D classes of selected particles for the generation of 3D maps, related to figure 2. (A) 2D class averages of nsEMPEM experiments with week 30 pAbs from rabbit 187-192 using GPCysRRLL-I53-50A pre-complexed with 8.9F (top) and GPCysR4-I53-50A pre-complexed with 18.5C (bottom). Classes shown are the selected classes that were selected for 3D classification, reclassified to 16 classes. **(B)** 2D class averages of nsEMPEM experiments with week 18 pAbs from rabbit 187 and 189 using GPCysRRLL-I53-50A pre-complexed with 8.9F. Classes shown are the selected classes that were selected for 3D classification, reclassified to 16 classes.

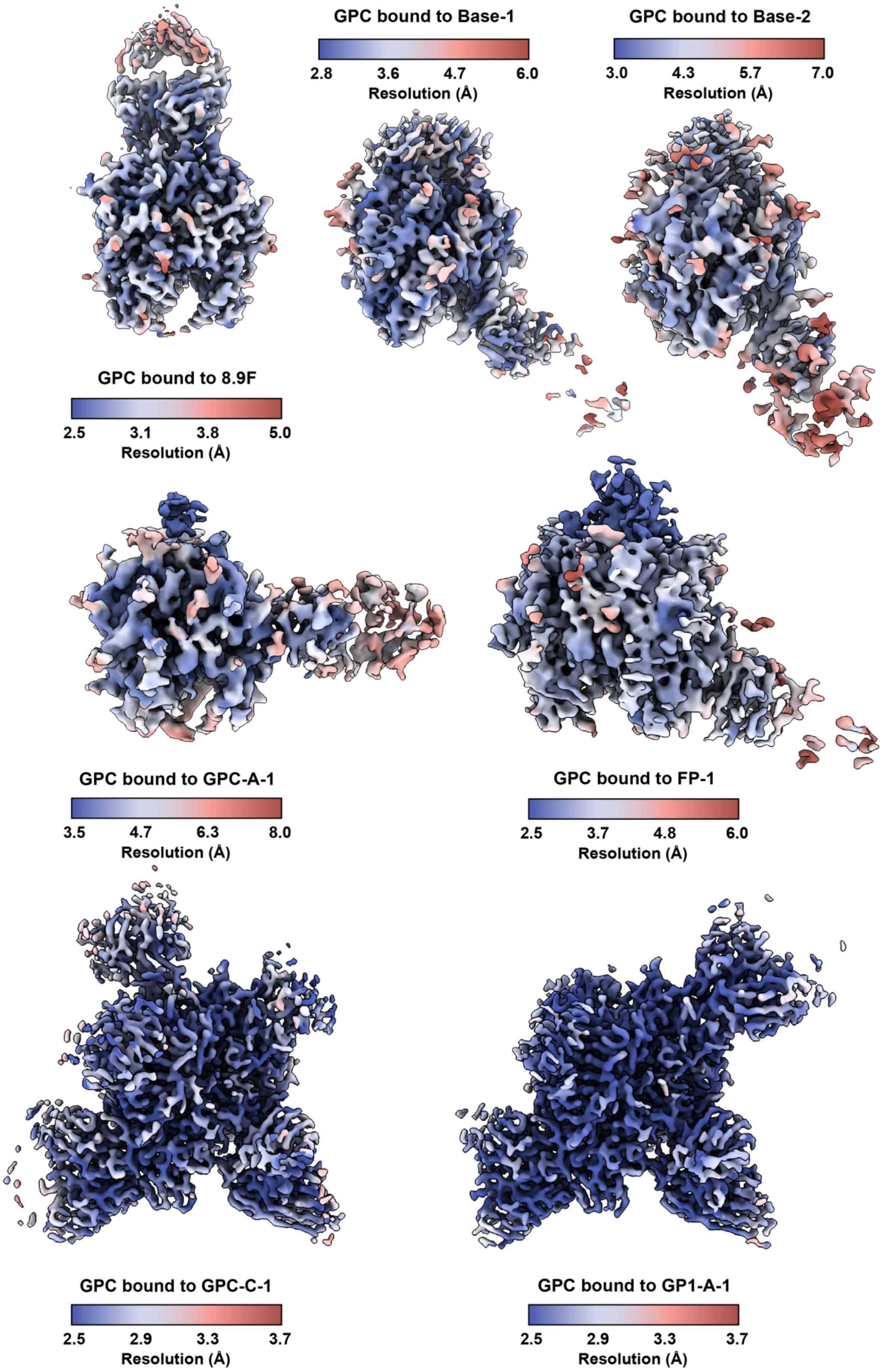

Fig. S3: Local resolution plots for EM maps of LASV GPC and 8.9F, Base-1, Base-2, GPC-A-1, FP-1, GPC-C-1, and GP1-A-1, related to figures 3, 4, and 6. Local resolutions calculated using cryoSPARC 3.2[2] using an FSC threshold of 0.143. Maps visualized in ChimeraX.[3]

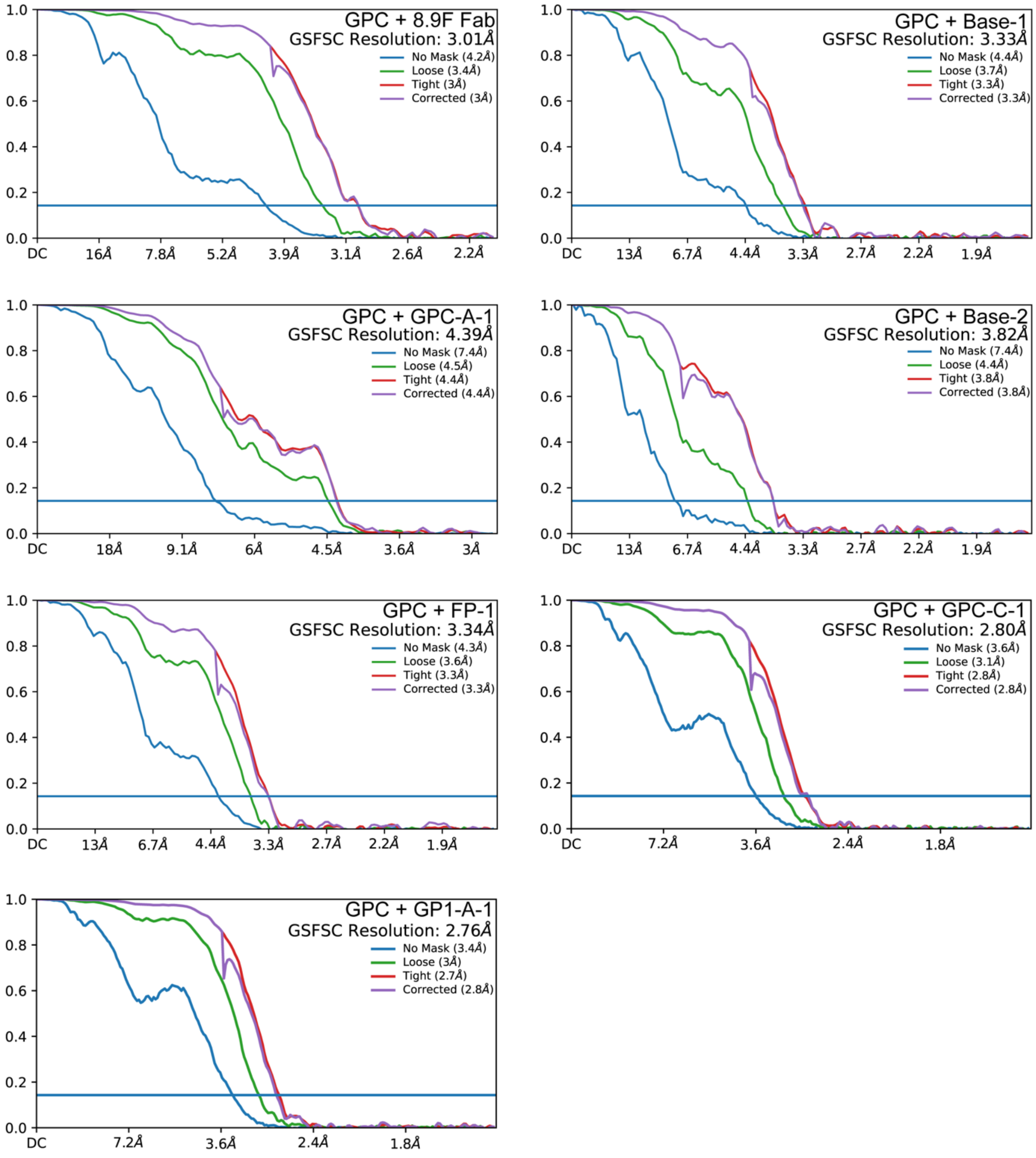

Fig. S4: FSC plots for cryo-EM maps, related to figures 3, 4, and 6. Reported resolutions coincide with an FSC cutoff of 0.143. Plots generated using cryoSPARC 3.2.[2]

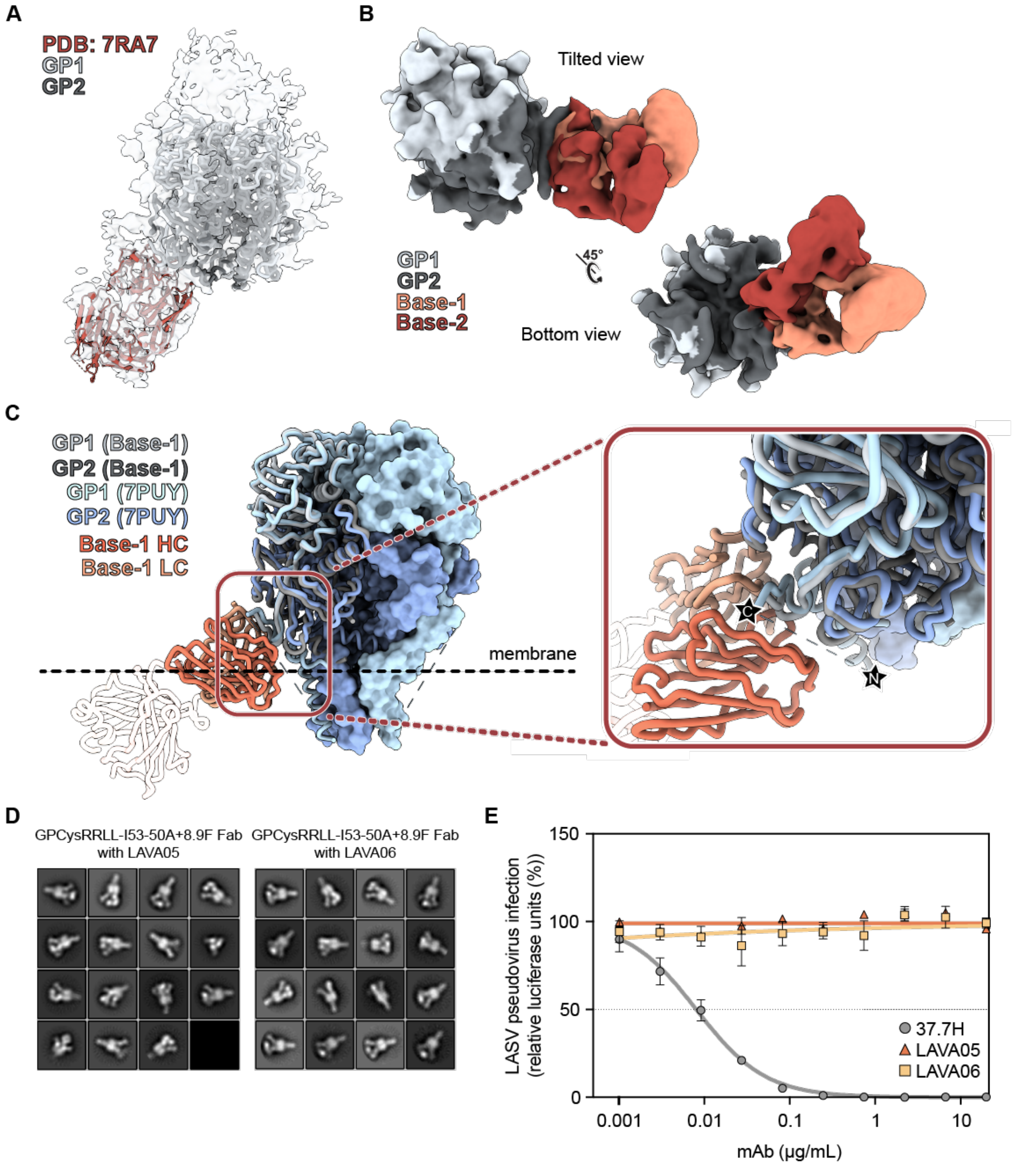

Fig. S5. Structural differences between Base-1 and Base-2 responses, their inability to bind membrane-embedded GPC and neutralization of LAVA05 and LAVA06, related to figure 3. (A) Cryo-EM map of Base-2 with a rabbit Fab (PDB: 7RA7) docked in the pFab density. **(B)** Overlaid Gaussian-filtered maps of base antibodies demonstrating the distinct angles of approach taken by Base-1 (orange) and Base-2 (red) to engage the GPC. **(C)** Overlay of full-length GPC (PDB: 7PUY[4]) with the Base-1 model revealing accessibility issues for Base-1 binding by the membrane and/or SSP. The constant region of the Fab (transparent) was generated by docking a model of a full rabbit Fab (PDB: 7RA7) into the density and removing the variable domain. The

inset shows the unmodelled part of the SSP (displayed as pseudobond), referred to by Katz et al.[4] as "hydrophobic 2" (residues 36-58), with its N terminus (right) and C terminus (left) depicted as stars.[4] It is probable that the 22 amino acid "hydrophobic 2" structure resides in this area, sterically blocking binding of base-targeting pAb responses. **(D)** 2D class averages of nsEMPEM experiments with LAVA05 and LAVA05 using GPCysRRLL-I53-50A pre-complexed with 8.9F. Classes shown are the selected classes that were selected for 3D classification, reclassified to 16 classes. **(E)** Pseudovirus neutralization of LAVA05 and LAVA06. The mAb 37.7H was used as a positive control.

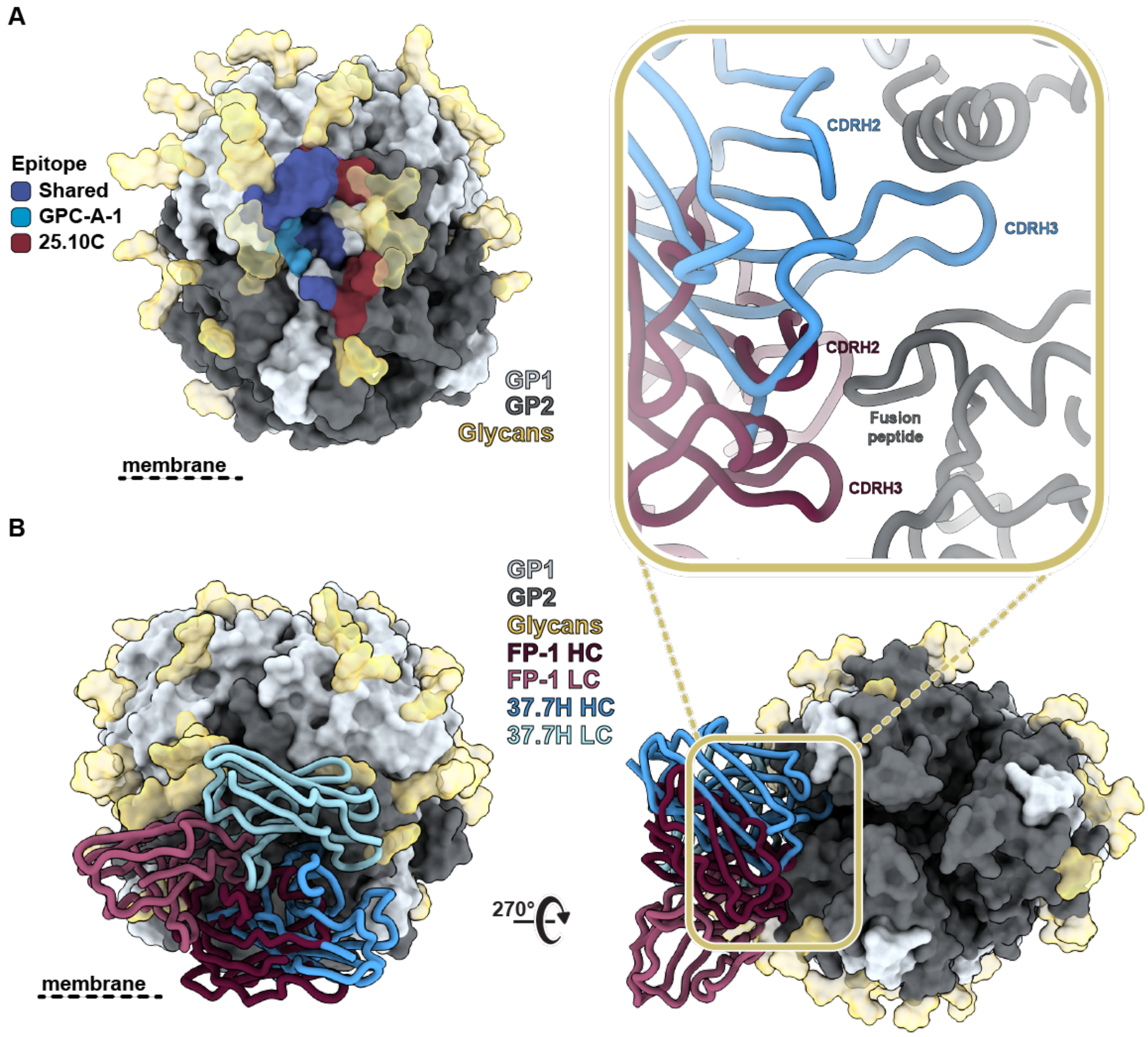

Figure S6. Comparison of GPC-A-1 and FP-1 with previously described human NAbs, related to figure 4. (A) Epitope footprints of GPC-A-1 and GPC-A NAb 25.10C.[5] Epitope-Analyzer[6] was used to determine (putative) contact residues using a cut-off of 4 Å between epitope and paratope for 25.10C and 6 Å for GPC-A-1 (poly-alanine model). **(B)** Comparison of GPC engagement by 37.7H and FP-1. Inset highlights the differences in fusion peptide engagement between the two antibodies.

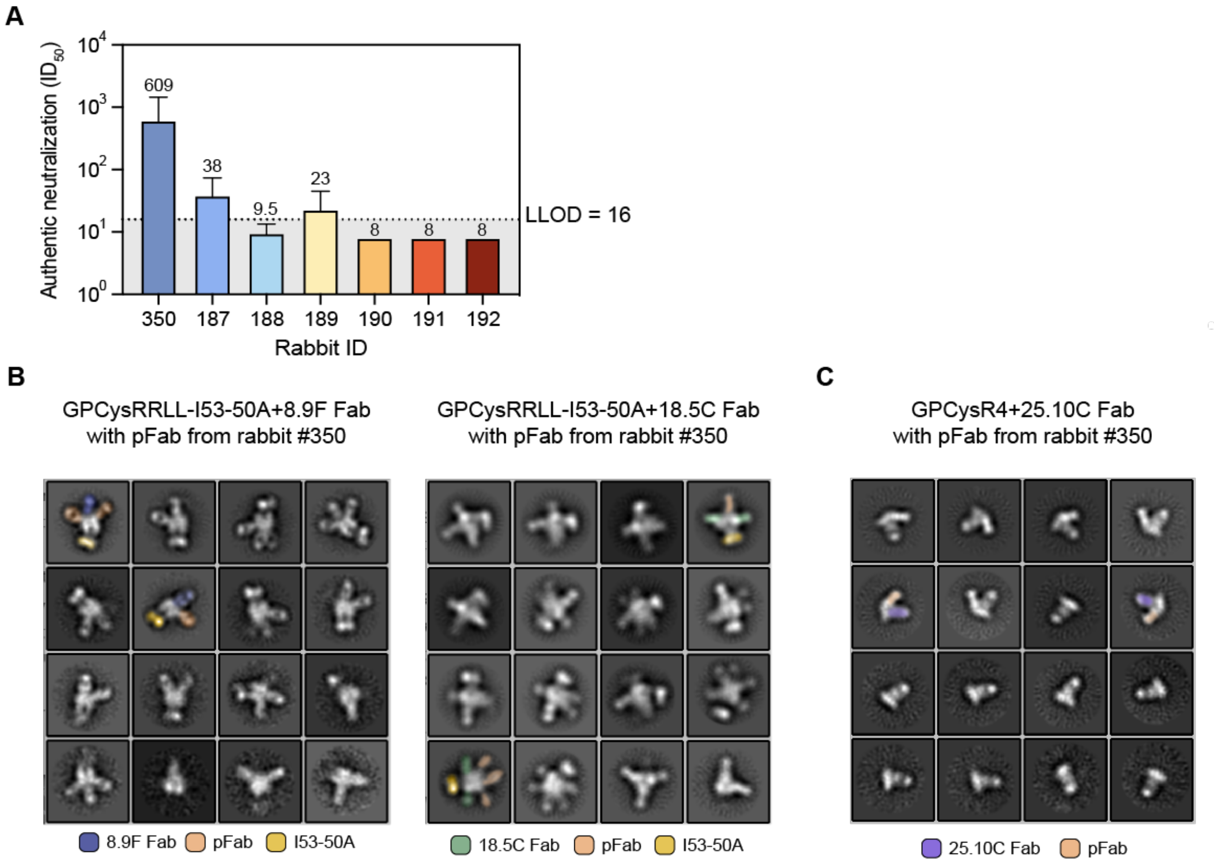

Figure S7. Comparison of neutralization titers to authentic LASV (Josiah strain) between GPCysR4-I53-50A-vaccinated rabbits and rabbit 350 and 2D class averages of nsEMPEM experiments with pAbs from rabbit 350, related to figure 6. (A) Autologous neutralization titers to authentic LASV from GPCysR4-I53-50A immunized rabbits (187-192) and a rabbit immunized with a GPC-derived VLP (rabbit 350). The virus neutralization titer is calculated as the geometric mean titers (GMT) of the reciprocal value of the last serum dilution at which inhibition of the cytopathic effect on infected Vero E6 cells is detectable. The initial dilution was 1:16 (LLOD) so a titer of 8 was noted when no inhibition was observed. Shown is the GMT of four technical replicates. **(B)** 2D class averages of nsEMPEM experiments with pAbs from rabbit 350 using GPCysRRLL-I53-50A pre-complexed with 8.9F (left) and 18.5C (right). Classes shown are the selected classes from two iterations of 2D classification that have been reclassified to 16 classes. Two classes are pseudo-colored according to the legend below to indicate the stabilizing Fab (8.9F Fab/18.5C Fab), pFab and I53-50A. **(C)** 2D class averages of monomeric GP nsEMPEM experiments with pAb from rabbit 350. Classes shown are the selected classes from two iterations of 2D classification that have been reclassified to 16 classes. Two classes are pseudo-colored according to the legend below to indicate the fiducial (25.10C Fab) and pFab.

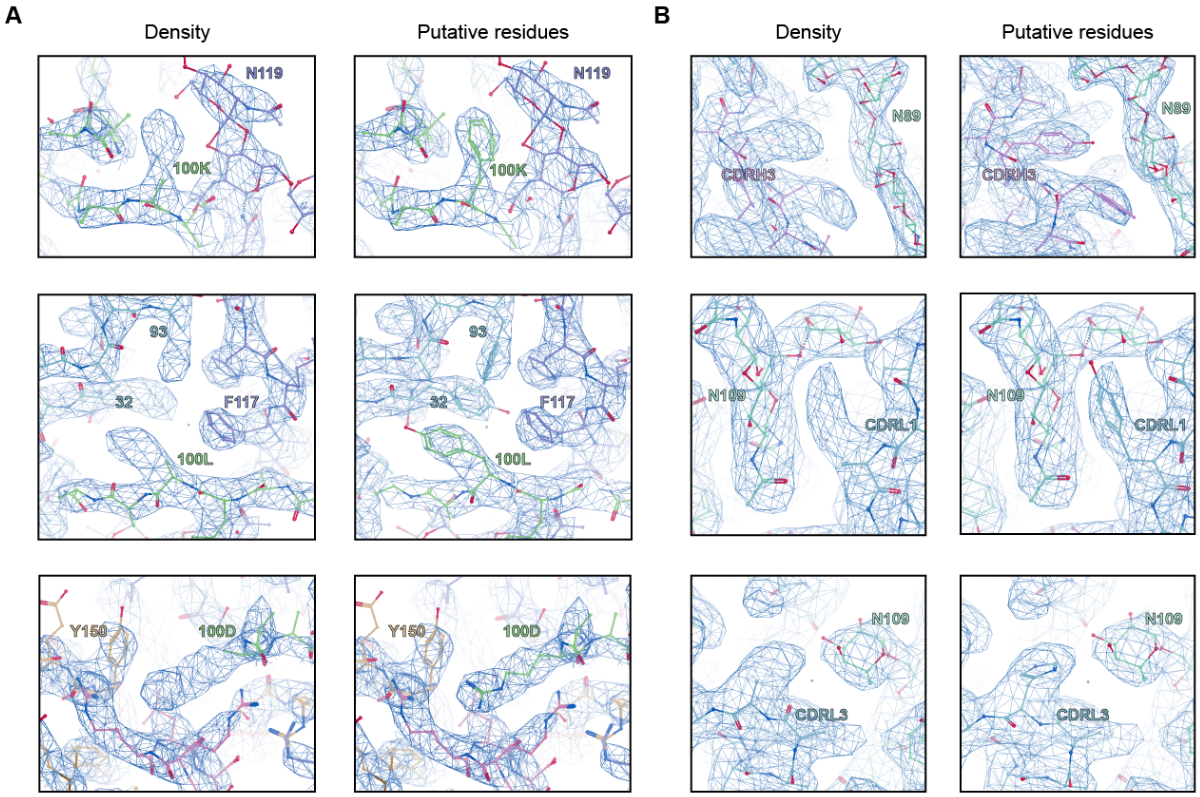

Figure S8. Clear side-chain densities that enable speculation of GPC-pFab interactions for GPC-C-1 (A) and GP1-A-1 (B), related to figure 6. (A) Aromatic density at position 100K in the CDRH3, making contacts with glycan N119 (top). Aromatic density at position 100L in the CDRH3, 32 in LC FR2, and and 93 in LC FR4 forming putative hydrophobic interactions with F117 in GPC (middle). Arginine density at positions 100A (not shown) and 100D in the CDRH3 presumably form cation-pi interactions with GPC's Y150 (bottom). **(B)** Aromatic densities in CDRH3 making contacts with the N89 glycan (top). Aromatic density in CDRL1 (middle) and CDRL3 (bottom) making contact with the N109 glycan.

Table S1. 8.9F antibody interactions with GP1, related to figure 1. Amino acid interactions at the 8.9F epitope-paratope region were determined using the online-based Epitope-Analyzer platform[6]. Glycan contacts were assessed by finding close contacts (<4 Å) of the GPC glycans with 8.9F Fab using ChimeraX[3].

GPC Residue number	Amino acid	Chain	Atom	Fab Residue number	Amino Acid	Heavy or light chain	Distance (Å)	Interaction Type
117	PHE	A	CD1-OH	100E	TYR	H	4.0	Van-der-Waals
119	ASN	A	OD1-CD1	100E	TYR	H	3.7	Van-der-Waals
121	SER	A	CB-CB	100D	TYR	H	3.8	Van-der-Waals
121	SER	A	OG-N	100D	TYR	H	3.9	Hydro-Bond
125	LYS	A	CE-OD2	100A	ASP	H	3.9	Van-der-Waals
125	LYS	A	NZ-OD1	100A	ASP	H	3.9	Salt-Bridge
125	LYS	A	NZ-OD2	100A	ASP	H	2.8	Salt-Bridge
150	TYR	A	CD2-O	100H	ASP	H	3.9	Van-der-Waals
150	TYR	A	CE1-CG2	100I	VAL	H	4.0	Hydr-Phbc
256	ARG	A	CB-OE1	100L	GLN	H	3.7	Van-der-Waals
256	ARG	A	CZ-CE	100G	MET	H	3.9	Van-der-Waals
256	ARG	A	NH2-O	100G	MET	H	3.2	Hydro-Bond
150	TYR	B	OH-OD2	100O	ASP	H	3.6	Hydro-Bond
256	ARG	B	CD-CE	100G	MET	H	3.1	Van-der-Waals
256	ARG	B	CD-CZ	100C	TYR	H	4.0	Van-der-Waals
257	ARG	B	CB-OH	100D	TYR	H	3.8	Van-der-Waals
257	ARG	B	N-OH	100D	TYR	H	3.7	Hydro-Bond
258	LEU	B	N-OH	100D	TYR	H	3.9	Hydro-Bond
125	LYS	C	CD-OD2	100O	ASP	H	3.6	Van-der-Waals
125	LYS	C	NZ-OD2	100O	ASP	H	2.8	Salt-Bridge
150	TYR	C	CE1-CB	100	ALA	H	3.6	Hydr-Phbc
150	TYR	C	CE2-OD1	100A	ASP	H	3.4	Van-der-Waals
150	TYR	C	CG-CE2	100D	TYR	H	3.9	Hydr-Phbc
150	TYR	C	CZ-CB	100	ALA	H	3.8	Hydr-Phbc
150	TYR	C	OH-OD1	100A	ASP	H	2.6	Hydro-Bond
150	TYR	C	OH-OD2	100A	ASP	H	3.8	Hydro-Bond
256	ARG	C	C-OD2	100H	ASP	H	3.7	Van-der-Waals
256	ARG	C	CD-CB	100G	MET	H	4.0	Van-der-Waals
257	ARG	C	C-OD1	100H	ASP	H	3.8	Van-der-Waals
257	ARG	C	N-OD1	100H	ASP	H	3.4	Hydro-Bond
257	ARG	C	N-OD2	100H	ASP	H	2.7	Hydro-Bond
258	LEU	C	CA-OD1	100H	ASP	H	3.8	Van-der-Waals
258	LEU	C	N-OD1	100H	ASP	H	2.9	Hydro-Bond
258	LEU	C	N-OD2	100H	ASP	H	4.0	Hydro-Bond
114	ASN	C	C-CA	30	GLY	L	3.7	Van-der-Waals
114	ASN	C	ND2-CB	30	ILE	L	3.9	Van-der-Waals
114	ASN	C	O-N	30	GLY	L	3.8	Hydro-Bond
115	HIS	C	CB-OD1	31	ASN	L	3.9	Van-der-Waals
116	LYS	C	N-CB	93	SER	L	4.0	Van-der-Waals
117	PHE	C	CE2-CG	31	ASN	L	3.8	Van-der-Waals
117	PHE	C	CZ-CA	93	SER	L	3.9	Van-der-Waals
117	PHE	C	CZ-CD2	91	TYR	L	3.9	Hydr-Phbc
117	PHE	C	CZ-CE2	32	PHE	L	3.9	Hydr-Phbc
117	PHE	C	CZ-CE2	91	TYR	L	3.9	Hydr-Phbc
N119 glycan, O4-C1 GlcNAc		A	N2-O	54	SER	H	3.0	-
N119 glycan, O4-C1 GlcNAc		A	C8-O	54	SER	H	3.7	-
N119 glycan, O6-C1 Fuc		A	O5-OG	54	SER	H	3.7	-
N119 glycan, O6-C1 Fuc		A	C1-O	54	SER	H	3.5	-
N119 glycan, O4-C1 GlcNAc		A	C7-O	54	SER	H	3.8	-
N119 glycan, O4-C1 GlcNAc		A	N2-C	54	SER	H	3.9	-
N119 glycan, O4-C1 GlcNAc		A	C2-O	54	SER	H	4.0	-
N119 glycan, O4-C1 GlcNAc		A	C8-CA	54	SER	H	4.0	-
N119 glycan, O6-C1 Fuc		A	O5-CB	54	SER	H	4.0	-

N119 glycan, O6-C1 Fuc	A	O4-CB	56	SER	H	3.2	-
N119 glycan, O6-C1 Fuc	A	O5-CB	56	SER	H	3.9	-
N119 glycan, O6-C1 Fuc	A	O4-CA	56	SER	H	4.0	-
N119 glycan, O6-C1 Man	A	O6-OG1	73	THR	H	2.9	-
N119 glycan, O6-C1 Man	A	O6-CB	73	THR	H	3.9	-
N119 glycan, O6-C1 Man	A	O6-N	73	THR	H	3.4	-
N119 glycan, O6-C1 Man	A	C6-OG1	73	THR	H	3.6	-
N119 glycan, O6-C1 Fuc	A	C6-O	100A	ASP	H	3.6	-
N119 glycan, O6-C1 Fuc	A	C6-OD1	100B	ASP	H	3.7	-
N119 glycan, ND1-C1 GlcNAc	A	C6-CZ	100E	TYR	H	3.8	-
N119 glycan, O4-C1 GlcNAc	A	C8-OH	100E	TYR	H	3.5	-
N119 glycan, ND1-C1 GlcNAc	A	C5-CE1	100E	TYR	H	3.7	-
N119 glycan, ND1-C1 GlcNAc	A	C6-CE1	100E	TYR	H	3.8	-
N119 glycan, ND1-C1 GlcNAc	A	O5-CE1	100E	TYR	H	3.8	-
N119 glycan, ND1-C1 GlcNAc	C	C8-O	100O	ASP	H	3.9	-
N119 glycan, ND1-C1 GlcNAc	C	C8-CD2	100Q	TYR	H	3.8	-
N119 glycan, ND1-C1 GlcNAc	C	O7-OH	100S	TYR	H	3.2	-
N119 glycan, ND1-C1 GlcNAc	C	O7-CZ	100S	TYR	H	3.8	-
N119 glycan, ND1-C1 GlcNAc	C	O7-CE2	100S	TYR	H	3.5	-
N119 glycan, O6-C1 Fuc	C	O4-OD1	31	ASN	L	2.8	-
N119 glycan, O6-C1 Fuc	C	C4-OD1	31	ASN	L	3.7	-
N119 glycan, O6-C1 Fuc	C	O4-CG	31	ASN	L	3.7	-
N119 glycan, O6-C1 Fuc	C	O3-C	31	ASN	L	3.7	-
N119 glycan, O6-C1 Fuc	C	O3-CA	31	ASN	L	3.7	-
N119 glycan, O6-C1 Fuc	C	O4-CB	31	ASN	L	3.8	-
N119 glycan, O6-C1 Fuc	C	C4-CB	31	ASN	L	3.9	-
N119 glycan, O6-C1 Fuc	C	O3-N	32	PHE	L	2.8	-
N119 glycan, O6-C1 Fuc	C	O3-CA	32	PHE	L	3.7	-
N119 glycan, O6-C1 Fuc	C	C5-CZ	32	PHE	L	3.8	-
N119 glycan, O6-C1 Fuc	C	C4-CE1	32	PHE	L	3.8	-
N119 glycan, O6-C1 Fuc	C	C4-CZ	32	PHE	L	3.9	-
N119 glycan, O6-C1 Fuc	C	C3-CD1	32	PHE	L	3.9	-
N119 glycan, O6-C1 Fuc	C	C3-CE1	32	PHE	L	3.9	-
N119 glycan, O6-C1 Fuc	C	C3-N	32	PHE	L	3.8	-
N119 glycan, O6-C1 Fuc	C	C5-CE1	32	PHE	L	3.9	-
N119 glycan, O6-C1 Fuc	C	C6-CZ	32	PHE	L	4.0	-
N119 glycan, ND1-C1 GlcNAc	C	O3-OE2	50	GLU	L	3.5	-
N119 glycan, ND1-C1 GlcNAc	C	O3-OE1	50	GLU	L	4.0	-
N119, O4-C1 GlcNAc	C	C1-OE1	50	GLU	L	3.7	-
N119 glycan, ND1-C1 GlcNAc	C	O3-CD	50	GLU	L	3.8	-
N119, O6-C1 Man	C	O6-CG1	51	VAL	L	3.5	-
N119, O6-C1 Man	C	O6-CB	51	VAL	L	3.9	-
N119, O4-C1 Man	C	O5-O	51	VAL	L	4.0	-
N119, O4-C1 Man	C	O2-CB	53	SER	L	3.4	-
N119, O4-C1 Man	C	O2-OG	53	SER	L	3.8	-
N119, O3-C1 Man	C	O3-OG	53	SER	L	3.2	-
N119, O4-C1 Man	C	O2-CA	53	SER	L	3.8	-
N119 glycan, O6-C1 Fuc	C	O3-CE	66	LYS	L	3.6	-
N119 glycan, O6-C1 Fuc	C	O3-CD	66	LYS	L	3.8	-
N119 glycan, O6-C1 Fuc	C	O3-NZ	66	LYS	L	3.9	-

Table S2: Electron Microscopy Data Bank deposition information, related to figures 2 and 3. Maps are accessible at emdataresource.org using the listed codes. Additional maps can be found on the “Download” tab of each entry.

	EMDB ID	Time point	Specificity	# particles (composite)	Map	Microscope	Pixel size
**Rabbit 187**	EMD-43174	30	Base	7,212	additional map	Tecnai Spirit	2.06
	EMD-43174	30	FP	10,038	additional map	Tecnai Spirit	2.06
	EMD-43174	30	GPC-A	16,689	main map	Tecnai Spirit	2.06
	EMD-43175	18	Base	8,882	additional map	Tecnai Spirit	2.06
	EMD-43175	18	FP	8,905	additional map	Tecnai Spirit	2.06
	EMD-43175	18	GPC-A	12,945	main map	Tecnai Spirit	2.06
**Rabbit 188**	EMD-43176	30	Base	9,484	main map	Tecnai Spirit	2.06
**Rabbit 189**	EMD-43177	30	Base	12,037	additional map	Tecnai Spirit	2.06
	EMD-43177	30	GPC-A	13,250	main map	Tecnai Spirit	2.06
	EMD-43178	18	Base	5,061	additional map	Tecnai Spirit	2.06
	EMD-43178	18	GPC-A	2,937	main map	Tecnai Spirit	2.06
**Rabbit 190**	EMD-43179	30	Base	6,805	additional map	Tecnai Spirit	2.06
	EMD-43179	30	FP	11,371	main map	Tecnai Spirit	2.06
**Rabbit 191**	EMD-43180	30	Base	11,393	main map	Tecnai Spirit	2.06
**Rabbit 192**	EMD-43181	30	Base	15,413	additional map	Tecnai Spirit	2.06
	EMD-43181	30	FP	15,136	main map	Tecnai Spirit	2.06
**LAVA05**	EMD-43182	30	Base	17,515	main map	Tecnai Spirit	2.06
**LAVA06**	EMD-43183	30	Base	19,440	main map	Tecnai Spirit	2.06

Table S3: Cryo-EM data collection and atomic model refinement, related to figures 3, 4, 5, and 6.

	GPC bound to mAb 8.9F	GPC bound to pAb GPC-A-1	GPC bound to pAb Base-1	GPC bound to pAb Base-2	GPC bound to pAb FP-1	GP monomer bound to INT-1 and INT-2	GPC bound to pAb GPC-C-1	GPC bound to pAb GP1-A-1
**Access codes**								
PDB	8ER3	8TYB	8TYC	NA	8TYE	N/A	8VCV	8VE8
EMDB	EMD-28549	EMD-41712	EMD-41713	EMD-41714	EMD-41715	EMD-41716	EMD-43141	EMD-43168
Genbank	NP_694870.1	NP_694870.1	NP_694870.1	NP_694870.1	NP_694870.1	NP_694870.1	NP_694870.1	NP_694870.1
**Data collection and processing**								
Microscope	Titan Krios	Titan Krios	Titan Krios	Titan Krios	Titan Krios	TFS Glacios	TFS Glacios II	TFS Glacios II
Magnification	130,000	130,000	130,000	130,000	130,000	190000	190000	190000
Voltage (kV)	300	300	300	300	300	200	200	200
Electron exposure (e^-/Å^2)	50.0	49.1	50.35	50.35	50.35	49.5	40.7	40.7
Defocus range (μm)	-0.7 to -2.0	-0.7 to -2.0	-0.7 to -2.0	-0.7 to -2.0	-0.7 to -2.0	-0.8 to -1.4	-0.8 to -2.0	-0.8 to -2.0
Pixel size (Å)	1.045	0.725	0.833	0.833	0.833	0.725	0.718	0.718
Imposed Symmetry	C1	C1	C1	C1	C1	C1	C1	C1
Final particle number	73,222	30,182 (symmetry expanded)	61,338 (symmetry expanded)	14,690 (symmetry expanded)	77,295 (symmetry expanded)	20,839	89,195	146,722
Map resolution (Å)	3.0	4.4	3.3	3.8	3.3	8.1	2.8	2.8
FSC Threshold	0.143	0.143	0.143	0.143	0.143	0.143	0.143	0.143
Map sharpening B-factor (Å^2)	-35	-96	-60	-80	-90	-896	-58	-64
**Model refinement and validation**								
Total Residues	1389	1369	1353		1351		1343	1329
Amino-acids	1300	1281	1273		1266		1267	1260
Carbohydrates	89	88	80		85		76	69
RMSD Bonds	0.02	0.02	0.02		0.02		0.02	0.02
RMSD Angles	1.7	1.8	1.7		1.8		1.7	1.8
**Ramachandran**								
Outliers (%)	0	0	0	N/A	0	N/A	0	0
Allowed (%)	2.12	4.38	4.48		3.79		2.89	2.75
Favored (%)	97.88	95.62	95.52		96.21		97.11	97.25
Rotamer outliers (%)	0	0	0		0		0	0
Clash score	2.1	3.1	3.1		3.1		3.2	2.7
Molprobity score	1.0	1.4	1.4		1.4		1.3	1.2
FSC model (0/0.143/0.5)	2.7/3.0/3.3	4.2/4.3/4.6	3.0/3.3/3.7		3.2/3.4/3.9		2.7/2.8/3.0	2.7/2.7/2.9
EMRinger score	4.58	1.42	2.83		2.09		5.5	5.55

References

1. Li, H., Buck, T., Zandonatti, M., Yin, J., Moon-Walker, A., Fang, J., Koval, A., Heinrich, M.L., Rowland, M.M., Diaz Avalos, R., et al. (2022). A cocktail of protective antibodies subverts the dense glycan shield of Lassa virus. Sci. Transl. Med. *14*, eabq0991.
2. Punjani, A., Rubinstein, J.L., Fleet, D.J., and Brubaker, M.A. (2017). cryoSPARC: algorithms for rapid unsupervised cryo-EM structure determination. Nat. Methods *14*, 290–296.
3. Pettersen, E.F., Goddard, T.D., Huang, C.C., Meng, E.C., Couch, G.S., Croll, T.I., Morris, J.H., and Ferrin, T.E. (2021). UCSF ChimeraX: Structure visualization for researchers, educators, and developers. Protein Sci. *30*, 70–82.
4. Katz, M., Weinstein, J., Eilon-Ashkenazy, M., Gehring, K., Cohen-Dvashi, H., Elad, N., Fleishman, S.J., and Diskin, R. (2022). Structure and receptor recognition by the Lassa virus spike complex. Nature *603*, 174–179.
5. Enriquez, A.S., Buck, T.K., Li, H., Norris, M.J., Moon-Walker, A., Zandonatti, M.A., Harkins, S.S., Robinson, J.E., Branco, L.M., Garry, R.F., et al. (2022). Delineating the mechanism of anti-Lassa virus GPC-A neutralizing antibodies. Cell Rep. *39*, 110841.
